# Neuropsychological Deficits Chronically Developed after Focal Ischemic Stroke and Beneficial Effects of Pharmacological Hypothermia in the Mouse

**DOI:** 10.14336/AD.2019.0507

**Published:** 2020-02-01

**Authors:** Weiwei Zhong, Yan Yuan, Xiaohuan Gu, Samuel In-young Kim, Ryan Chin, Modupe Loye, Thomas A Dix, Ling Wei, Shan Ping Yu

**Affiliations:** ^1^Department of Anesthesiology, Emory University School of Medicine, Atlanta, GA 30322, USA.; ^2^Center for Visual and Neurocognitive Rehabilitation, Atlanta Veterans Affairs Medical Center, Decatur, GA 30033, USA.; ^3^College of Veterinary Medicine, Yangzhou University, Yangzhou, 225009, China.; ^4^Department of Drug Discovery and Biomedical Sciences, Medical University of South Carolina, Charleston, SC 29401, USA.

**Keywords:** stroke, pharmacological hypothermia, post-stroke depression (PSD), BDNF, oxytocin

## Abstract

Stroke is a leading cause of human death and disability, with around 30% of stroke patients develop neuropsychological/neuropsychiatric symptoms, such as post-stroke depression (PSD). Basic and translational research on post-stroke psychological disorders is limited. In a focal ischemic stroke mouse model with selective damage to the sensorimotor cortex, sensorimotor deficits develop soon after stroke and spontaneous recovery is observed in 2-4 weeks. We identified that mice subjected to a focal ischemic insult gradually developed depression/anxiety like behaviors 4 to 8 weeks after stroke. Psychological/psychiatric disorders were revealed in multiple behavioral examinations, including the forced swim, tail suspension, sucrose preference, and open field tests. Altered neuronal plasticity such as suppressed long-term potentiation (LTP), reduced BDNF and oxytocin signaling, and disturbed dopamine synthesis/uptake were detected in the prefrontal cortex (PFC) during the chronic phase after stroke. Pharmacological hypothermia induced by the neurotensin receptor 1 (NTR1) agonist HPI-363 was applied as an acute treatment after stroke. A six-hr hypothermia treatment applied 45 min after stroke prevented depression and anxiety like behaviors examined at 6 weeks after stroke, as well as restored BDNF expression and oxytocin signaling. Additionally, hypothermia induced by physical cooling also showed an anti-depression and anti-anxiety effect. The data suggested a delayed beneficial effect of acute hypothermia treatment on chronically developed post-stroke neuropsychological disorders, associated with regulation of synaptic plasticity, neurotrophic factors, dopaminergic activity, and oxytocin signaling in the PFC.

Stroke remains a serious threat to human life and health. Up to 2/3 of stroke survivors suffer from functional disabilities. Additionally, they are at high risk of developing psychological/psychiatric disabilities, such as post-stroke anxiety, depression, and cognitive deficits [1]. Among these stroke patients, around 22% of patients experience anxiety at 3 months post-stroke [2] and over 1/3 of patients develop depression within 5 years of a stroke attack [3]. Post-stroke anxiety is closely associated with post-stroke depression (PSD) [4]. While post-stroke neuropsychological disorders largely affect outcomes of stroke patients, there has been limited research and few effective treatments available for PSD and other psychologic/psychiatric problems.

Some previous investigations examined post-stroke anxiety and PSD in rodent stroke models. In a mouse stroke model of 30-min middle cerebral artery (MCA) occlusion, depression like behaviors in the sucrose preference test and forced swim test, and anxious-like behaviors in the elevated plus maze test were observed at 14 weeks after the ischemic insult [5]. In the permanent MCA occlusion model of rats, similar phenotypes were detected at 3 weeks post-stroke [6]. Both stroke models involve occlusion of the main MCA branch and the formation of a large infarct that includes sub-cortical structures. Whether a small stroke restricted to cortical tissues could result in delayed psychological alterations is obscure. The small stroke model of mice induced by ligations of distal branches of the right MCA selectively damages the right sensorimotor cortex and is well known for its resultant sensorimotor deficits, specifically the dysfunction of the whisker barrel pathway [7-9]. In this investigation, we report chronically developed neuropsychological deficits after the small cortical ischemic stroke.

Antidepressants, such as selective serotonin reuptake inhibitors (SSRIs), have been suggested to treat stroke survivors with depression and anxiety symptoms [10]. However, their efficacies are far from satisfactory, especially in the elderly [11, 12]. Increased risks of hemorrhagic complication and sides effects on the gastrointestinal tract are concerns as well [4]. Therapeutic hypothermia, also known as targeted temperature management, has been demonstrated as a potential acute protective therapy after brain injuries. Emerging evidence from pre-clinical and clinical studies have shown that therapeutic hypothermia is a promising protective acute therapy for stroke. In our previous studies, we developed a pharmacological hypothermia treatment using neurotensin receptor 1 (NTR1) agonists and demonstrated the brain protective effects against ischemic stroke, hemorrhage stroke, and traumatic brain injury (TBI) [13, 14]. The pharmacologically induced hypothermia applied in the acute phase after stroke reduces infarct size and enhances functional recovery, which are mediated by multiple mechanisms including the attenuation of apoptosis, autophagy, regulation of inflammatory activities, blood brain barrier protection, and growth factor regulation [13-16]. Nonetheless, how therapeutic hypothermia in the acute phase after stroke may show impacts on chronically developed neuropsychological disorders such as PSD after stroke remains unknown.

In the present study, we performed a series of behavioral assessments to investigate the delayed effects of pharmacological hypothermia against chronically developed post-stroke neuropsychological deficits in the focal ischemic stroke mouse. Electrophysiology and molecular experiments were used to explore underlying mechanisms in the post-stroke cortex. Therapeutic hypothermia induced by NTR1 agonists provides acute protection in the ischemic core and peri-infarct regions through the inhibition of multiple injurious pathways such as oxidative stress, cell death signaling, and inflammatory responses [13-16]. We now provide new evidence for prolonging benefits of the hypothermia therapy on post-stroke psychological disabilities by regulating inflammation, secondary neurodegeneration, and synaptic plasticity in the ischemic and non-ischemic brain regions.

## MATERIALS AND METHODS

### Animals

Adult male C57BL/6 mice aged 3-5 months old were used in this study. The mice were housed in standard cages in 12-hr light/12-hr dark cycle in the Emory University animal facility where the room temperature was kept at 22±1°C. All experimental procedures were conducted in accordance with the National Institutes of Health (NIH) Guide for the Care and Use of Laboratory Animals and were approved by the Emory University Institutional Animal Care and Use Committee. The animals used in the study were randomly divided into controls and experimental groups.

### Focal Cerebral Ischemic Stroke Model of Mice

Focal cerebral ischemic stroke targeting the right sensorimotor cortex was induced by occlusion of the distal branch of the middle cerebral artery (MCA), as described previously [14]. Briefly, anesthesia was induced using 3.5% isoflurane followed by the maintenance dose of 1.5% isoflurane. A 10-mm incision was made midway between the right eye and ear. A 4-mm diameter circle was incised in the skull and the encircled bone was avulsed. The transparent dura was left intact. The cortex ischemia was achieved by permanent occlusion of the distal branch of the right middle cerebral artery (MCA) supplying the sensorimotor cortex. The MCA occlusion was paired with 7-min ligation of both common carotid arteries (CCAs). Sham control mice only received surgery lesions on the skull surface.

### Brain Slice Preparation

At indicated post-stroke days, mice were decapitated after deep anesthesia with inhalation of saturated isoflurane. The forebrain was obtained and immediately placed in an ice-cold and sucrose-rich aCSF (in mM) containing 220 sucrose, 10 d-glucose, 1.9 KCl, 6 MgCl_2_, 0.5 CaCl_2_, 1.2 NaH_2_PO_4_ and 33 NaHCO_3_. The solution was bubbled with 95% O_2_ balanced with 5% CO_2_ (pH 7.40). The transverse pontine sections (~350 μm) containing the barrel cortex were obtained using a vibratome sectioning system, and then recovered at 33 °C for 60min in normal aCSF (in mM) containing 10 d-glucose, 124 NaCl, 3 KCl, 2 MgCl_2_, 2 CaCl_2_, 1.3 NaH_2_PO_4_ and 26 NaHCO_3_. The slices were kept at room temperature before use. At recording, a slice was transferred to the MEA dish that was perfused with oxygenated aCSF at a rate of 8 ml/min and maintained at 34 °C in a recording chamber by a dual automatic temperature control (Warner Instruments).

### Microelectrode Array (MEA) Recording

A high-resolution MEA2100-system (MultiChannel Systems, Reutlingen, Germany) was used to record the field excitatory postsynaptic potential (fEPSP) in brain slices. The MEA chamber (60pMEA200/30iR-Ti, MultiChannel Systems GmbH, Reutlingen, Germany) is composed of a 6-mm high glass ring and an 8x8 Titanium nitride electrode grid (59 electrodes and 1 internal reference electrode) with an electrode diameter of 30 μm and spacing of 200 μm. The brain slice was transferred to the MEA chamber that was perfused with oxygenated aCSF at a rate of 6-8ml/min and maintained at 34°C for 10 mins before recording. During recording, electric stimuli (±1.5 V, 10 ms) were applied every 30 sec and the evoked fEPSPs were simultaneously monitored in different locations of the brain slices. For LTP recording, baseline responses were recorded for 10-15?min for stabilization. In the amygdala, fEPSPs were recorded in the basolateral amygdala (BLA) with the stimulation in lateral amygdala (LA) and LTP was induced by a series of high-frequency stimulation (100 Hz, 1 sec, 5 trains with 1 min interval). In the PFC, fEPSPs were recorded in L2/3 with stimulation in L5 and LTP was induced by applying a high-frequency stimulation (100 Hz, 1sec, 2 trains with 30s interval). The slopes of fEPSPs were analyzed offline with Multi-Channel Analyzer V 2.6.0 (Multichannel Systems) and GraphPad Prism 6 (GraphPad Software, San Diego, CA, USA).

### RT-PCR of RNA measurements

Total RNAs from the brain tissue containing the right prefrontal cortex (PFC) were isolated and cDNAs were synthesized with the high-capacity cDNA reverse transcription kit (Life Technologies). Quantitative PCR (qPCR) was performed with Fast SYBR Green Master Mix (Applied Biosystems, Life Technologies), following the instructions of the manufacturer, in a fast-real-time PCR system (Applied Biosystems 7500) for 40 cycles. PCR primers were as follows: 18s (forward, GTAACCCGTTGAACCCCATT; reverse, CCATCCAA TCGGTAGTAGCG), NR1 (forward, TACAAGCGAC ACAAGGATGC; reverse, TCAGTGGGATGGTACTG CTG), NR2A (forward, CTGCTCCAGTTTGTTGGTG A; reverse, AGATGCCCGTAAGCCACA), NR2B (forward, GGGTTACAACCGGTGCCTA; reverse, CTT TGCCGATGGTGAAAGAT), GluR1 (forward, CGGA AATTGCTTATGGGACA; reverse, ACACAGCGATT TTAGACCTCCT), TNF-α (forward, GGAACACGTCG TGGGATAATG; reverse, GGCAGACTTTGGATGC TTCTT), IL-1β (forward, TCGGCCAAGACAGGTCG CTCA; reverse, TGGTTGCCCATCAGAGGCAAGG), IL-6 (forward, TAGTCCTTCCTACCCCAATTTCC; reverse: TTGGTCCTTAGCCACTCCTTC) and IL-10 subunit (forward, CCCATTCCTCGTCACGATCTC; reverse, TCAGACTGGTTTGGGATAGGTTT). 18 s was used as the internal control for the quantification of expression.

### Western Blot analysis of protein expression

The brain tissue of the right prefrontal cortex was processed in a lysis buffer containing (in mM) 20 Na_4_P_2_O_7_, 10 Tris-HCl (pH 7.4), 100 NaCl, 1 EDTA (pH 8.0), 1 EGTA, 2 Na_3_VO_4_, 1% Triton, 1% protease inhibitor cocktail (Sigma-Aldrich, St. Louis, MO) and 1% protease inhibitor. BCA protein assay reagent (Pierce Biotechnology, Rockford, IL) was used to estimate the protein concentration, and 30 g of proteins were used to detect targeted signals in SDS-PAGE gels and electrophoretically transferred to a PVDF membrane. The membranes were then blocked with 5% bovine serum albumin for 1 hour and incubated overnight at 4 °C with primary antibodies. The primary antibodies used and the dilutions for each were mouse β-actin (Sigma-Aldrich) at 1:5000, rabbit tubulin (Cell Signaling, Danvers, MA) at 1:1000, rabbit NR1 (Millipore, Burlington, MA) at 1:600, rabbit NR2A (Millipore) at 1:1000, mouse NR2B (Millipore) at 1:1000, rabbit GluR1 (Abcam, Cambridge, MA) at 1: 1000, rabbit BDNF (Santa Cruz Biotech, Inc., Dallas, TX) at 1:500, rabbit Oxytocin (Fitzgerald Industries International, North Acton, MA) at 1:1000, rabbit Oxytocin Receptor (Abcam) at 1:1000, rabbit Myelin Basic Protein (MBP) (Abcam) at1:1000, rabbit Synaptophysin (Cell Signaling) at 1:1000, rabbit PSD95 (Cell Signaling) at 1:1000, rabbit goat DAT (Santa Cruz Biotech) at 1:500, rabbit TH (Millipore) at 1:1000, rabbit GAD65/67 (Millipore) at 1:500, rabbit 5HT2AR (Abcam) at 1:100, rabbit TNF-α (Cell Signaling) at 1:1000, rabbit IL-1β (Cell Signaling) at 1:1000, rabbit IL-6 (Cell Signaling) at 1:1000, mouse anti-IL-10 (Santa Cruz Biotechnology) at 1:500. After washing in TBST, the membranes were incubated with AP-conjugated secondary antibodies (GE Healthcare, Piscataway, NJ, USA) for 1 h at room temperature. The bromo-chloroidolylphosphate/nitroblue tetrazolium (BCIP/NBP) solution (Sigma-Aldrich) was used to detect signals and the photographs were scanned. The immunoblotting signals were then quantified using ImageJ software (NIH, Baltimore, MD).

### Induction of pharmacological hypothermia using HPI-363

Pharmacological hypothermia was induced by the NTR1 agonist HPI-363 as described before [14, 16]. A rectal probe (Harvard Apparatus, Holliston, MA) was used to measure the rectal temperatures of experimental animals. The body temperature was maintained at 36.5 ± 1.0°C during surgery using a heating pad controlled by a homeothermic blanket control unit (Harvard Apparatus). After surgery, mice were allowed to recover in a humidity-controlled incubator (Thermocare, Incline Village, NV). The experimental mice were randomly assigned into four groups: sham, stroke, stroke with hypothermia induced by HPI-363, and stroke with hypothermia induced by physical cooling. Animals in sham and stroke groups were injected with saline after surgery and their body temperatures were maintained at 36.0 ± 1.0°C in a humidity-controlled incubator for 6 hours. Animals in hypothermia groups were subjected either to HPI363 injections or ice exposure (4°C). HPI363 was dissolved in saline and injected intraperitoneally into the experimental mice. The first bolus injection (2 mg/kg) was given at 45 min after CCA reperfusion (52 min after the onset of MCA occlusion), followed by additional injections at half the initial dose (1 mg/kg). The interval between the injections was ≥ 2 hours, with adjustments made to keep a constant mild hypothermia between 32~34°C for 6 hrs. For animals in the physical cooling group, animals were placed in a mouse holder covered by ice for the first 5 min and then in ≥4°C chamber to maintain the body temperature at 32~34°C for 6 hrs. The Physitemp temperature monitoring system (Physitemp Instruments, Inc., Clifton, NJ) allowed simultaneous monitoring and data acquisition from seven animals during or after anesthesia.

### Sucrose Preference Test

The sucrose preference test was used to detect the depression like behavior of anhedonia. Briefly, two weighed bottles containing ~40 ml tap water were placed in each cage and mice had free access to bottles for 3 days. After an adaptation period, the mice were given free access to one bottle containing 30 ml of tap water and another containing 30 ml of 1% sucrose water (w/v). Twenty-four hrs later, sucrose preference was calculated using the following formula: sucrose consumption (g)?/?(water consumption (g) + sucrose consumption (g))?×?100% [17, 18]. During the sucrose preference test, the animals were individually housed. When returning to their home cages, they were socially housed with maximum 5 animals per cage.

### Forced Swim Test

The forced swim test was conducted to evaluate the depression like behaviors of mice. All experimental mice were habituated in the waiting room for at least 30 min before testing. In the test, a cylinder of 18?cm in diameter and 30?cm in height was filled with water at ~25°C.The experimental mouse was placed in the water and the swim activity was video recorded for 6?min. The swim and floating behavior during the last 4 min were analyzed. The mice were considered as immobile when passively floating and exhibiting movements necessary only to keep the nose above water [19].

### Tail Suspension Test

The tail suspension test was used to test depression like behaviors. All experimental mice were habituated in the waiting room for at least 30 min before testing. In the test, mice were suspended by their tails with adhesive tape in a position about 1?cm away from the tail tips. The tests were video recorded for 6?min and the last 5 min were analyzed. Immobility was considered only if the mice completely stopped any initiated movements [20].

### Open Field Test

The open field test was performed to evaluate the anxious behavior of mice. In the tests, each animal was introduced in the center of an open arena sized 50?cm?×?50?cm × 50?cm (L × W × T) and allowed to move freely for 10?min. Behaviors, such as travel distances and locations, were recorded using TopScan CleverSys (Clever Sys, Inc.) and the videos were analyzed by the TopScan Realtime Option Version 3.0 (Clever Sys Inc.). The arena was cleaned with 70% ethanol after each test.

### Statistics

The behavioral tests and data analyses were done double-blindly by two to three people without information of grouping. The sample sizes were tested with G-Power Analysis to yield sufficient statistical power. GraphPad Prism 6 (GraphPad Software) was used for statistical analysis and graphic presentation. The electrophysiological data were analyzed with Multi-Channel Analyzer (Multi Channel Systems). Data are presented as mean ± S.E. Student’s t-test was used for two groups comparison. For the comparisons within multiple groups one-way ANOVA was applied when there was only one independent variable and two-way ANOVA was applied when there were two independent variables. Fisher's post hoc test was used to perform further analyses. The difference was considered significant when p < 0.05.

**Figure 1.**
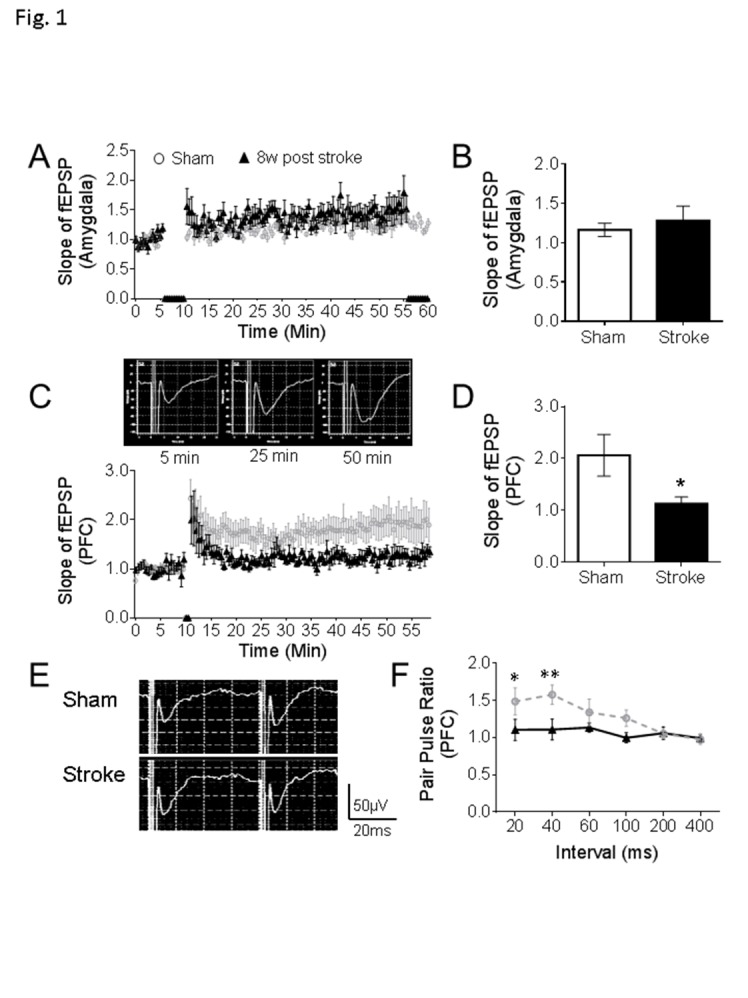
**Defects of synaptic plasticity in the prefrontal cortex after focal ischemic stroke**. **(A-B)** In the amygdala, stable fEPSPs were recorded for 6 min as a baseline, high frequency stimulation (HFS) induced an LTP-like response, but no significant differences were found between stroke mice and sham (sham: n = 8, stroke: n = 9). **(C-D)** In the PFC of sham mice, HFS initiated the LTP as displayed in the representative traces. In the comparison to sham animals, smaller LTPs were recorded in the PFC of mice at 8 weeks after stroke (C). At 50 min of the recording, the slope of fEPSP was significantly reduced in the PFC of stroke mice than sham controls (D). **(E-F)** Representative traces are pair-pulse response with a 40 ms interval in PFC. Stroke insult notably reduced the pair-pulse ratio of fEPSPs at 20 ms and 40 ms intervals (Sham: n = 8, Stroke: n = 9; *P < 0.05, **P < 0.01; Student’s *t*-test).

## RESULTS

### Impaired synaptic plasticity in the prefrontal cortex after focal ischemic stroke

A small stroke model of right sensorimotor cortex ischemia with partial reperfusion in adult mice was tested in this investigation [14, 21-23]. The ischemic insult included the permanent occlusion of the distal branch of the right MCA, accompanied with 7-min ligation of the bilateral CCAs. The focal cortical ischemia and the partial reperfusion mimic many clinical cases of small strokes that often show spontaneous or therapy (chemical/ thrombectomy)-induced reperfusion [7]. The stroke model exhibits a restricted focal infarction in the sensorimotor cortex, followed by secondary selective neuronal damage in the thalamus without detectable pathological injury to other brain regions [24]. Whether pathological alterations could occur in non-injured regions, especially in the brain structures associated with psychological behaviors is an open question and remains to be directly evaluated.

**Figure 2.**
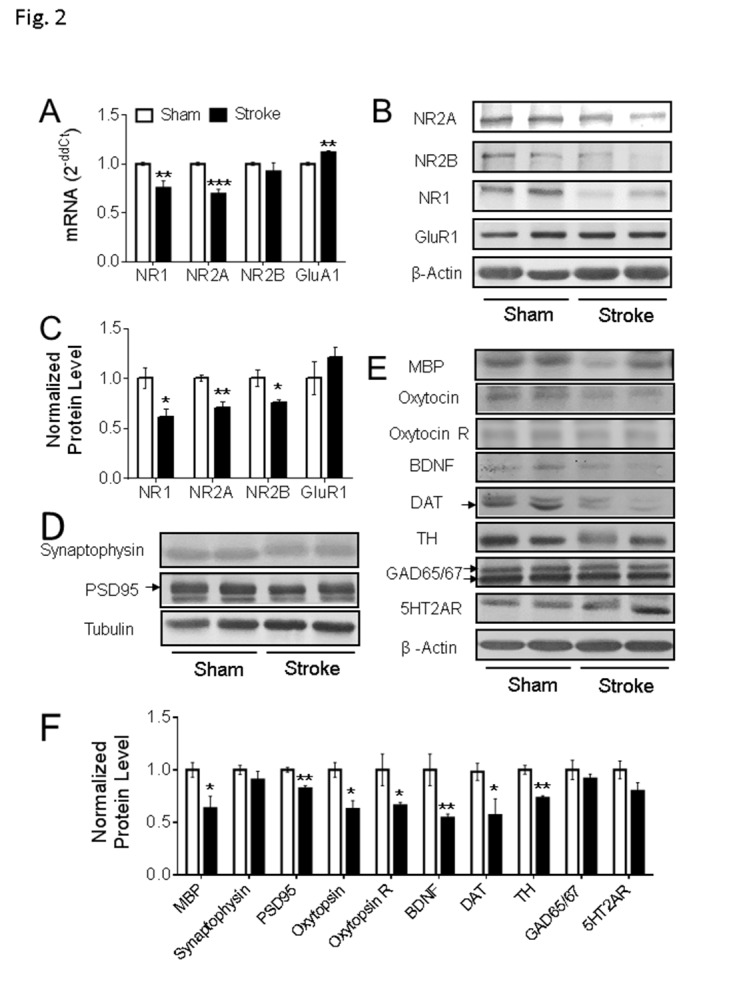
**Gene expression in the PFC of focal ischemic stroke mice**. **(A-C)** In the ipsilateral PFC of the animals with stroke for 8 weeks, qPCR data suggested the mRNA level of NR1 and NR2A were significantly reduced (A, Sham: n = 5, Stroke: n = 5), which is consistent with the western blot results (B-C, Sham: n = 4, Stroke: n = 4). The mRNA level of GluR1 was significantly increased (A), although no significant difference was detected in the protein expression level of GluR1 (B-C) (*P < 0.05, **P < 0.01, ***P < 0.001; Student’s t-test). **(D-F)** In the western blot assessment, compared to the animals in the sham group, the protein expression levels of MBP, PSD95, oxytocin, oxytocin R, BDNF, DAT, and TH were significantly reduced in PFC of stroke animals. Arrows point to the bands of PSD95 and DAT; the two GAD65/67 bands were used in the quantification. (Sham: n = 4, Stroke: n = 5; *P < 0.05, **P < 0.01; Student’s t-test).

Multiple brain regions may be involved in depression/anxiety like behaviors, notably including amygdala and the prefrontal cortex (PFC) [25]. Since region specific alterations in synaptic plasticity such as long-term potentiation (LTP) have been linked to the development of depression [26-28], LTPs in the amygdala and PFC were recorded in the mouse brain slices using the Multiple Electrode Array (MEA) system. In the brain slice containing the amygdala, field excitatory postsynaptic potential (fEPSP) was recorded in the basolateral amygdala (BLA), which consists of the lateral, basal, and accessory-basal nuclei of the amygdala. The lateral nuclei receive most of the sensory information directly from the temporal lobe structures, including the hippocampus and primary auditory cortex. In the recording, the high frequency stimuli (HFS) was applied to the lateral amygdala (LA). Following the stable fEPSPs recording for 6 min, a series of HFS (100 Hz, 1 sec, 5 trains with 1 min interval) induced a small but persistent potentiation of fEPSPs in the normal or sham control brain slices (Fig. 1A). At 8 weeks post-stroke, the small LTP remained and no significant difference was detected between sham and stroke groups (Fig. 1A and 1B).

In the brain slice containing the PFC, fEPSPs were recorded at layer 2/3 with electric stimulation at layer 5 and LTP was induced by two trains of HFS (100 Hz, 1 sec) at 30 sec intervals. Compared to the LTP in the sham slices, a much weaker LTP was observed in the stroke group. The slope ratio of fEPSP was significantly smaller than that in sham animals (Fig. 1C, 1D and 1E). Meanwhile, we observed the altered pair-pulse ratios measured with 20 ms and 40 ms intervals in this region, indicating an impaired regulation of presynaptic neurotransmitter release (Fig. 1F and 1G). These results provide an electrophysiological background in two relevant brain regions and a potential mechanism that may associate with depression and anxiety like behaviors.

### Alterations of glutamatergic and dopaminergic synaptic systems, and neurotrophic signaling in the PFC after focal ischemic stroke

To further investigate the abnormalities in prefrontal synaptic plasticity, a series of cellular and molecular assessments were conducted. No TUNEL-positive cells were detected in the PFC at 3 days and 8 weeks after stroke, suggesting no detectable primary or secondary cell death in this non-injured region previously subjected to a brief non-lethal hypoperfusion (i.e. 7 min CCA occlusion) (Supplementary Fig 1). qPCR and Western blot were used to quantify the expression level of glutamate receptor subunits, including the NMDA receptor family NR1 (GluN1), NR2A (GluN2A), NR2B (GluN2B), and AMAP receptor GluR1. In mice with stroke for 8 weeks, both mRNA and protein levels of NR1 and NR2A were significantly reduced, accompanied with increased mRNA expression of GluR1 and reduced protein level of NR2B in the PFC (Fig. 2A - 2C). Coincidently, protein levels of myelin binding protein (MBP), a biomarker of myelination, and PSD95, a specific postsynaptic marker, were also significantly reduced (Fig 2D - 2F). These observations revealed significant changes in the synapse of the seemingly undamaged PFC at 8 weeks after focal ischemic stroke.

To further evaluate the possible pathogenic events in the PFC, Western blotting was used to explore the expression level of other key proteins associated with neuronal activities. Interestingly, the expression levels of oxytocin, oxytocin receptor, and BDNF were significantly decreased in the PFC examined at 8 weeks after stroke (Fig. 2E and 2F). Moreover, significantly reduced expressions of tyrosine hydroxylase (TH), the rate-limiting enzyme in dopamine synthesis, and dopamine transporter (DAT), a key factor in dopamine reuptake, were observed in the PFC of stroke mice (Fig. 2E and 2F). There was no significant difference between sham and stroke groups in the levels of GAD65/67, the rate-limiting enzyme in GABA synthesis, and 5HT2AR, a key serotonin receptor involved in the emotion regulation (Fig. 2E and 2F). These data indicated that the focal ischemic insult occurred 8 weeks earlier imposed marked selective disturbances on the expression of certain genes that are key players in synaptic plasticity, neurotrophic signaling, and dopamine regulation in a functionally related brain region.

### Chronically developed psychological behaviors with impaired neuronal plasticity in the prefrontal cortex

**Figure 3.**
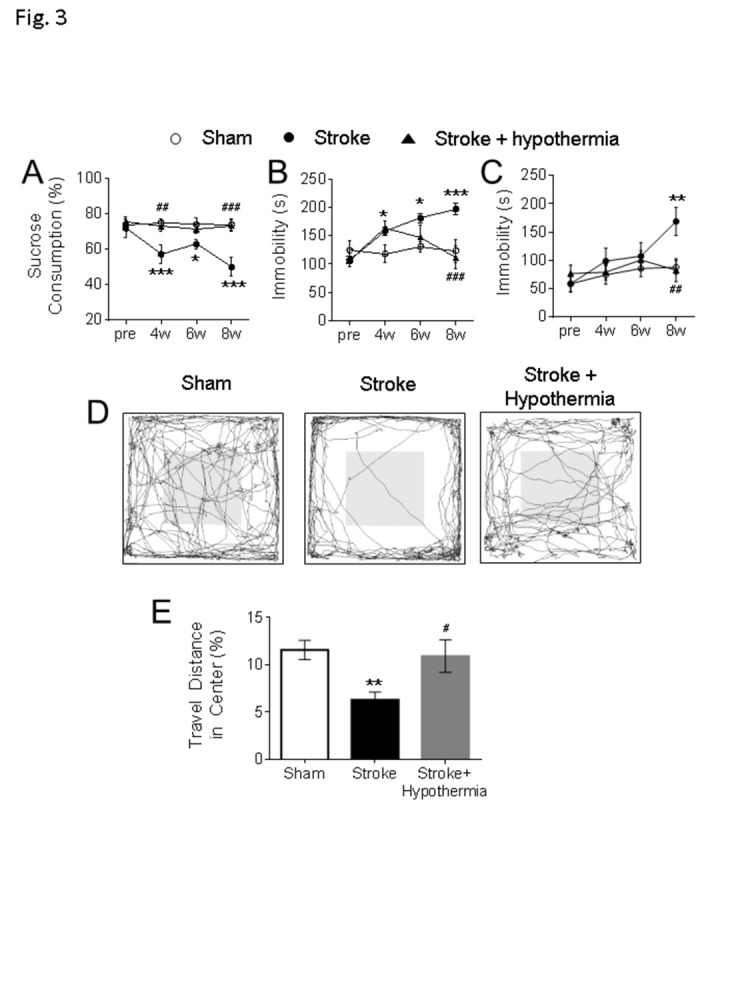
**Delayed development of neuropsychological behaviors and beneficial effects of pharmacological hypothermia after focal ischemic stroke**. **(A)** The sucrose preference test was used to test anhedonic behavior in mice. In comparison to the sham, the stroke animals consumed significantly less sucrose 2-8 weeks post stroke and acute hypothermia treatment significantly increased the sucrose consumption at 4 weeks and 8 weeks post stroke. The main factor of treatment showed a significant difference (F=16.3, df =2, P < 0.001). No significant differences were found in the main factor of time (F = 2.24, df = 3, P = 0.088) or the interactions of time × treatment (F = 1.90, df = 6, P = 0.088). **(B-C)** The tail suspension (B) and forced swim test (C) were used to test the depression like behavior in mice. **(B)** In the tail suspension test, the stroke mice showed significantly increased immobile durations at 4-8 weeks after stroke and acute hypothermia treatment significantly reduced the immobility at 6 weeks and 8 weeks after stroke. Significant differences were detected in the main factor of time (F = 6.01, df = 3, P < 0.001), treatment (F = 11.24, df = 2, P < 0.001) and interaction of time × treatment (F = 4.79, df = 6, P < 0.001). **(C)** In the forced swim test, the stroke mice were immobile significantly longer than the sham and acute hypothermia treatment significantly reduced the immobility in stroke mice. There are significant differences in the main factor of time (F = 3.90, df = 3, P < 0.05) and treatment (F = 3.60, df = 2, P < 0.05). No significant difference was found in the interaction of time × treatment (F = 1.55, df = 6, P = 0.172) (D-E) The open field test was used to test the anxious-like behavior in mice. The walking traces of the test mice in the open field were shown as a representative (D). The animals traveled significantly less in the central region of the arena than sham at 6-7 weeks after stroke, while acute hypothermia treatment increased the ratio of traveling in the center region in these mice (E). (A-E, sham: n = 10, stroke: n = 10, stroke + hypothermia: n = 8) (* sham vs stroke; # stroke vs stroke + hypothermia; *, # P < 0.05, **, ## P < 0.01, ***, ### P < 0.001; Two-way ANOVA and One-way ANOVA with Fisher’s post hoc)

After the focal sensorimotor cortical ischemia, mice with this stroke developed sensorimotor deficits soon after the ischemic insult, followed by a gradual spontaneous recovery within 2-4 weeks [29]. To assess psychological changes, behavioral tests were performed to evaluate depression like behaviors before and after stroke. No significant depression like behaviors were detected at 2 weeks post stroke in the tail suspension and forced swim tests. (Fig. 3A-3C). In the sucrose preference test, animals normally consume ~75% of sucrose water, but they started to lose sucrose preference from 2 weeks after stroke; this behavior persisted at least 8 weeks after stroke (Fig. 3A). In the tail suspension test and forced swim test, significant increases in immobility time were detected from 4 weeks after stroke (Fig. 3B and 3C). Stroke animals also developed anxious-like behaviors in the open field test at 6 weeks after stroke by showing significantly less walking distances in the central region of the testing arena (Fig. 3D and 3E). Collectively, these animals with a small ischemic attack to the right sensorimotor cortex gradually developed psychological abnormalities at 2-8 weeks post stroke, significantly later than the onset of sensorimotor deficits and emerged after the spontaneous recovery of sensorimotor function.

**Figure 4.**
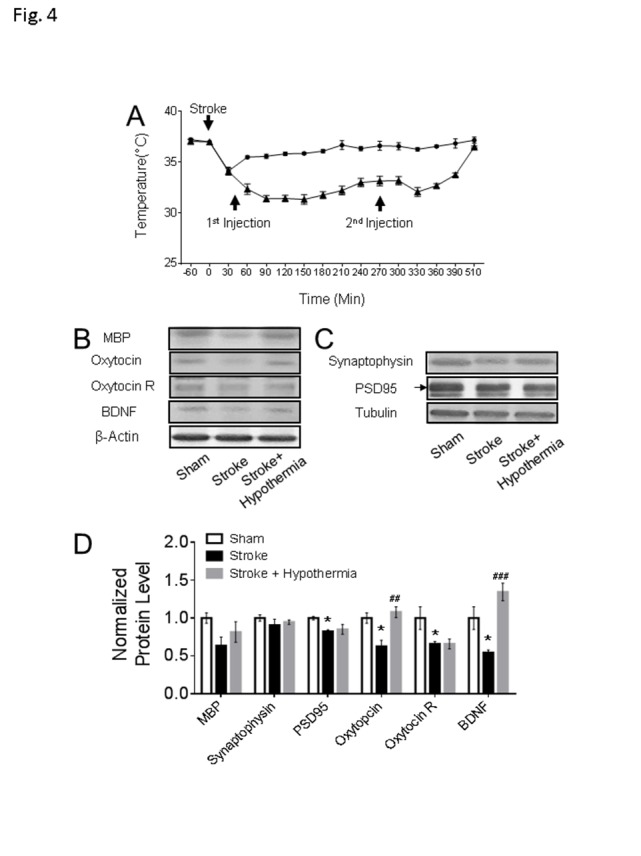
**Chronic consequences in the non-ischemic PFC after acute pharmacological hypothermia in focal ischemic stroke mice**. **(A)** The time course of pharmacologically induced hypothermia with HPI363 in mice after stroke (stroke: n = 10, stroke + hypothermia: n = 8). **(B-D)** The western blot results showed both the expression levels of oxytocin and BDNF were significantly increased in the stroke mice with hypothermia treatment. Only the active form of BDNF band and the arrow pointed PSD95 band were used in the quantification. (sham: n = 4, stroke: n = 5, stroke + hypothermia: n = 4) (* sham vs stroke; # stroke vs stroke + hypothermia; * P < 0.05, **, ## P < 0.01, ***, ### P < 0.001; One-way ANOVA with Fisher’s post hoc)

### Acute treatment of pharmacological hypothermia alleviated delayed psychological deficits after focal ischemic stroke

Pre-clinical and clinical studies have shown that therapeutic hypothermia is a promising acute protective therapy after strokes [15]; meanwhile, its potential effects on post-stroke psychological symptoms have rarely been examined. In the present investigation, we inspected the potential antagonism of the acutely administered hypothermic drug HPI-363 on psychological disorders chronically developed after stroke. HIP-363, as a NT analog, mediates hypothermia via activation of NTR1, which is densely expressed in the hypothalamus [30, 31].

Moderate hypothermia (~32-34°C) was achieved by a bolus injection of HPI-363 (2 mg/kg, i.p.) given at 45 min after the onset of ischemia. The low body temperature was maintained for 6 hrs by an additional injection at half of the initial dose (I mg/kg, i.p.) (Fig. 4A). We showed in previous reports that this treatment is protective against ischemic acute and subacute brain damage via suppression of inflammation, apoptosis/autophagy, gene regulation and blood brain barrier protection [13-15]. In the present experiment of 6-8 weeks after stroke, the acute hypothermia treatment attenuated the declined sucrose consumption in the sucrose preference test (Fig. 3A), the prolonged immobile duration in the tail suspension test, and forced swim test (Fig 3B, C). Meanwhile, the travel distance in the central arena was increased in the open field test of hypothermia treated stroke mice (Fig 3D). Consistently, Western blotting showed that protein expression levels of MBP, oxytocin, and BDNF were significantly increased in the post-ischemic brain of hypothermia group compared to stroke control group (Fig 4B and 4C).

**Figure 5.**
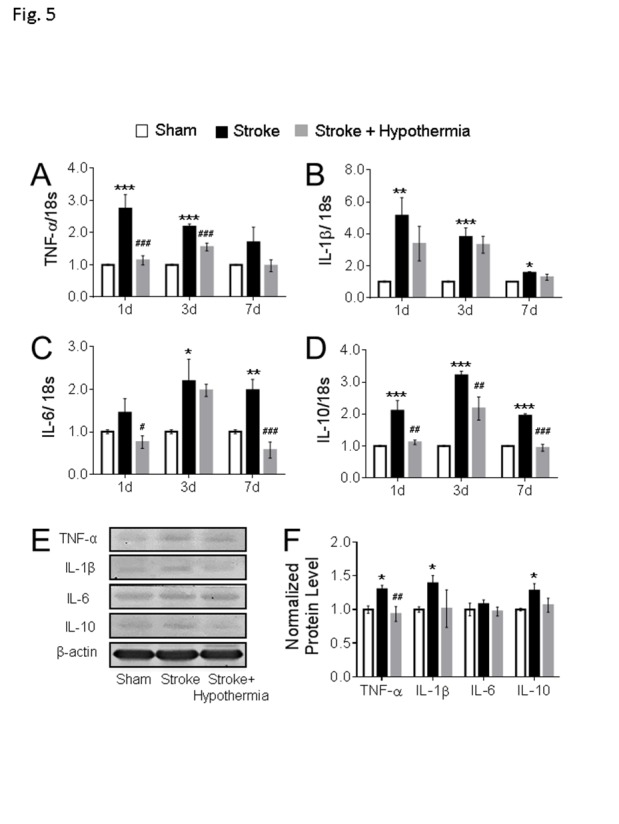
**Acute hypothermia treatment altered the cytokines expression in the non-ischemic PFC**. **(A-D)** The mRNA levels of cytokines were measured using qPCR analyses in the ipsilateral PFC at 1day, 3days and 7days after stroke. Stroke induction significantly enhanced the mRNAs of the pro-inflammatory cytokines TNF-α (A), IL-1β (B) and IL-6 (C) at 1day, 3day, and 7days post stroke. Hypothermia treatment significantly attenuated the TNF-α (A) at 1day and 3days post stroke and IL-6 at 1day and 7days post stroke. On the contrary, the mRNA of anti-inflammatory factor IL-10 (D) was significantly increased in stroke mice and hypothermia treatment reduced the IL-10 mRNA level in stroke mice at 1 day, 3 day and 7 days post-surgery. (n = 3 in each groups) (E-F) Western blot was used to quantify the cytokine levels in PFC at 3 days post stroke. Stroke induction enhanced the protein expression of TNF-α, IL-1β, and IL-10, while acute hypothermia treatment significantly reduced the TNF-α expression level in PFC at 3 days post stroke. No change was observed in the pro-inflammation cytokine IL-6 between groups. Only the active form of IL-1β was used in the quantification. (sham: n = 4, stroke: n = 4, stroke + hypothermia: n = 4) (* sham vs stroke; # stroke vs stroke + hypothermia; *, # P < 0.05, **, ## P < 0.01, ***, ### P < 0.001; One-way ANOVA with Fisher’s post hoc)

### Inflammatory activity in the PFC of stroke mice and effects of pharmacological hypothermia

Cerebral ischemia induces inflammatory reactions, such as the activation of glial cells, leukocytes, and release of pro-inflammatory cytokines [32, 33]. These may act as mediators in the pathogenesis of secondary injury after stroke; however, inflammation in non-injured regions such as the PFC has not been specifically examined. qPCR and Western blot measured the expression level of some key inflammatory mediators in the PFC. The mRNA levels of proinflammatory factors, including tumor necrosis factor-α (TNF-α), interleukin-1β (IL-1β) and interleukin-6 (IL-6) were significantly increased 1 to 7 days after stroke (Fig. 5A-5C). The mRNA level of the anti-inflammation cytokine IL-10 was also notably enhanced (Fig. 5D). In the Western blot assessment, the protein levels of TNF-α and IL-1β were increased, whereas no change was detected in IL-6 and IL-10 protein levels in the stroke mice at 3 days after stroke (Fig. 5E and 5F). These observations implicated that although the PFC was not a region directly attacked by the focal cerebral ischemia, it still exhibits immune responses with disruptions of inflammatory factors.

The inhibition of pro-inflammatory mediator production has been demonstrated to prevent brain injuries after stroke [34]. Our previous study showed that acute hypothermia treatment after stroke significantly ameliorated the inflammation in the ischemic region, leading to reduced brain injury in the stroke mice [14]. The impact of hypothermia on inflammation in the non-injured PFC was inspected in the present study. In stroke mice, the hypothermia treatment markedly reduced the mRNA level of pro-inflammatory factors TNF-α and IL-6 at 1 to 3 days after stroke and IL-6 l at 1 and 7 days after stroke, respectively (Fig. 5A and 5C). The mRNA level of anti-inflammatory IL-10 was reduced at 1, 3, and 7 days after stroke as well (Fig. 5D). In the Western blot assessment, the hypothermia treatment reduced the protein level of TNF-α at 3 days after surgery. No changes were found in other inflammatory factors (Fig. 5E and 5F). These data demonstrate that the early treatment of pharmacological hypothermia regulated the inflammatory responses, with an overall anti-inflammation profile in the non-injured PFC after focal ischemic stroke.

**Figure 6.**
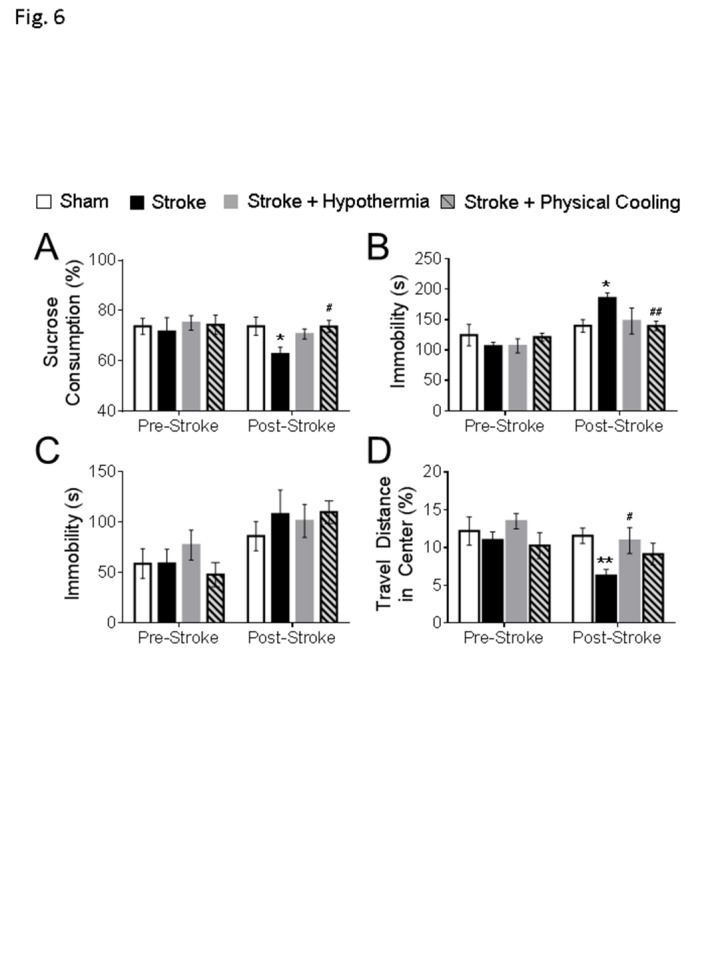
**Physical cooling alleviated psychological behaviors in mice after stroke**. The behavioral assessments were performed at 6-7 weeks post stroke in the experimental mice. **(A)** In the sucrose preference test, physical cooling prevented the loss of sucrose preference to levels similar to the drug induced hypothermia group. **(B)** In the tail suspension test, physical cooling significantly attenuated the immobility in stroke mice. **(C)** No significant differences were detected between groups in the forced swim test. **(D)** In the open field test, although physical cooling failed to significantly increase the travel distance ratio in the center, no differences were found between sham and stroke mice with physical cooling treatment. (sham: n = 10, stroke: n = 10, stroke + hypothermia: n = 8, stroke + physical cooling: n = 8; * sham vs stroke; # stroke vs stroke + hypothermia; *, # P < 0.05, **, ## P < 0.01; One-way ANOVA with Fisher’s post hoc)

### Physical cooling induced hypothermia alleviated the anxiety/depression like behaviors in mice after stroke

Activation of NTR1 results in an analgesic effect, which has been applied in the treatment of psychiatric disorders such as schizophrenia [30]. To verify the role of hypothermia in the observed anti-psychological effects observed with the NTR1 agonist, behavior assessments were repeated in stroke animals received an acute physical cooling treatment. Moderate hypothermia (32-34^o^C) was induced 45 min after stroke using ice and a cold environment and was maintained for 6 hrs after stroke to be consistent with the effects of pharmacological hypothermia. Six to seven weeks after stroke, the sucrose preference test revealed that physical cooling significantly increased the sucrose consumption in stroke mice (Fig. 6A). In the tail suspension test, the immobile durations were significantly reduced in the stroke mice with physical cooling treatment compared to stroke control animals (Fig. 6B). No differences were detected in the forced swim test between groups (Fig. 6C). In the open field test, the travel distances in the center arena did not show a significant difference between the stroke and physical cooling treated mice. However, there was also no significant difference between animals in the sham and physical cooling groups (Fig. 6D). Therefore, treatment of physical cooling acutely after stroke appeared to attenuate the development of psychological disorders. On the other hand, less significant effects with physical cooling seem to suggest that pharmacological hypothermia possesses stronger anti-psychological benefits possibly due to a better controlled cooling action and/or some direct beneficial effects of NTR1 activation.

## DISCUSSION

Post-stroke depression/anxiety is common neuro-psychological consequences of ischemic strokes. The present investigation is the first study to specifically evaluate neuropsychological disorders after a focal ischemic insult and to test the preventive action of an acute hypothermic treatment. In previous studies, we demonstrated acute brain protective effects of NTR1 agonist-induced hypothermia in brain injuries [13-15, 35-37]. The development of chronic neuropsychological and cognitive deficits after brain injuries such as ischemic stroke has been a focus of research interests in recent years. It is still obscure, however, whether a relatively small/mild ischemic insult may consequently lead to neuropsychological/neuropsychiatric deficits. Moreover, abnormal events at cellular and molecular levels in non-ischemic brain regions, e.g. PFC, associated with a specific behavior have rarely been investigated before. Various risk factors have been associated with the development of post-stroke neuropsychological/ neuropsychiatric disorders, such as age, medical history, genetic background, lesion location and damage features [11, 38]. The stroke size and damage severity have also been indicated as a significant factor with PSD occurrence. In this study, multiple cellular, molecular and behavioral assessments were performed at different time points after focal cortical ischemic stroke in mice. Although no cell death was detected in the PFC, significant psychological symptoms including anxious and depression like behaviors were detected 1 to 2 months after stroke accompanied with full recovery of sensorimotor functions.

Electrophysiological recordings and molecular analyses reveal delayed impairments in neuronal/synaptic plasticity, reduced neurotrophic signaling, and disrupted dopamine metabolic activities in the non-injured PFC. Furthermore, an acute treatment of 6-hr hypothermia applied soon after stroke showed an alleviation on the development of delayed depression and anxiety like symptoms. The time of the hypothermic treatment was 45 min after stroke, and we demonstrated previously that the therapeutic window of neuroprotective effects of the NRR1 compounds was around 2 hrs after the insult [37]. Considering that the hypothermic compounds are neuroprotective against both ischemic and hemorrhage strokes [35], their administration can be initiated early without the need for the identification of the type of stroke, an early treatment of this pharmacological hypothermia within 1-2 hrs after the onset of an stroke attack is clinically feasible.

The suppression on inflammation and improved expression of related genes in the PFC are suggested to be possible mechanisms mediating the anti-psychological effects. These results provide new evidence that NTR1 agonist-induced hypothermia not only provides acute neuroprotective effects, but also delayed benefits of the prevention and attenuation of post-stroke neuropsychological deficits. Indeed, therapeutic hypothermia has been applied in a series of clinical trials for post-resuscitation care in cardiac arrest patients and incorporated in the American Heart Association guidelines for over 10 years [39]. Thus, both pre-clinical and clinical evidence suggests a clinical translation possibility of pharmacologically induced hypothermia as a duel treatment of neuroprotection and anti-neuropsychological disorders.

The PFC region is widely thought to mediate emotional processing, and region-specific changes in synaptic function are associated with chronic stress and depression [40]. Basic research suggests that neuronal circuits involving BLA affect different anxious behaviors [41]. Additionally, both clinical and pre-clinical studies demonstrate the important role of reciprocal connections between the amygdala and PFC in the regulation of anxiety [25, 40, 42, 43]. The MRI data from stroke patients provide supports that disruptions in thalamocortical and/or PFC pathways may contribute to the occurrence of PSD [44, 45], and Vahid-Ansari F et al. demonstrated stroke injuries in PFC led to the persistent anxiety/depression like phenotypes in lab mice [46]. In the present investigation of the focal cortical stroke model, the PFC was not a typical ischemic region and no significant cell death of TUNEL positive cells was observed in this region after stroke. However, secondary neurodegeneration caused by the focal ischemia has been reported in remote brain regions [38]. For example, in severe MCA occlusion rodent models, secondary neuronal cell death was found in the substantia nigra and thalamus [47, 48]. We showed before that retrograde cell death in the thalamus occurs after the focal sensorimotor cortical stroke [24]. Another study reported disrupted excitability of surviving thalamocortical circuits after a focal brain injury in the sensorimotor cortex [49]. Indeed, the reduced MBP expression we identified in the PFC suggests a possible degeneration of axonal myelination in this region. These observations collectively support the idea that cellular and synaptic disruptions may take place in a remote but functionally connected brain area after a focal ischemic stroke, consequently impairing chronic functional outcomes.

Neuroinflammation and immune activation in the brain are associated with psychological/psychiatric symptoms, especially depression [50]. Clinical studies also show that pro-inflammatory cytokines, e.g. TNF-α, IL-1β, IL-6, CCL-2, and IL-18, increase in patients with depression or anxiety [51-55]. Although IL-6 and IL-1β have been reported to be neuro reparative after stroke by promotes post-stroke angiogenesis [56] and reduce glutamate mediated excitotoxic cell death [57], their neurodestructive roles are widely demonstrated. Besides, increased IL-6 serum level may act as a key factor in the onset of neuropsychiatric symptoms, including depression like phenotypes [58]. In our study, the increase in pro-inflammatory factors TNF-α, IL-1β and IL-6 in the PFC is an important observation for the mechanism of psychological symptoms. The increase in anti-inflammatory cytokine IL-10 is expected as a compensatory mechanism. However, the overwhelming activation of multiple pro-inflammatory factors likely dominates the inflammatory reaction in the post-stroke brain. It is possible that increased inflammation processes in the PFC can contribute to secondary neurodegeneration such as axonal demyelination and synaptic dysfunction, leading to the psychological symptoms gradually developed after stroke.

Clinical and basic research have demonstrated a strong association between low serum BDNF level and depressive symptoms in human and rodents [59]. In stroke patients in particular, chronically low circulating BDNF levels are correlated with the incidence of PSD [60, 61]. In our study, reduced BDNF levels were detected in the PFC at 8 weeks after stroke, which is consistent with the onset of the anxiety/depression like phenotypes. Importantly, a hypothermia treatment upregulated the BDNF level in these stroke mice. In addition, the oxytocin levels were significantly lower in the PFC of stroke mice with PSD symptoms while the hypothermia treatment restored oxytocin expression and normal behaviors. Oxytocin is a neuropeptide that acts as a social hormone in the CNS [22]. Administration of oxytocin has been demonstrated to restore social activities in autistic animals and to ameliorate emotional-social deficits associated with post-traumatic stress disorder (PTSD) [62]. Therefore, the enhanced BDNF and oxytocin signaling after the hypothermia treatment are likely important contributors to the belated and lasting anti-psychological benefits after stroke.

Neurotensin has been extensively studied in the past due to its strong association with dopaminergic modulation. It was reported that neurotensin induced hypothermia might be dependent on the interaction between dopamine circuits and neurotensin signaling in the CNS [63]. On the other hand, neurotensin displayed a potent analgesic effect in vivo [64]. This effect of neurotensin involves both NTR1 and neurotensin receptor 2 (NTR2), but the hypothermia effect is exclusively dependent on NTR1. As an analog of NT [8-13], HPI-363 is a specific agonist on NTR1 [30, 65]. To identify whether the effect observed with HPI-363 was due to the cooling action or other effects of NTR1 activation, physical cooling was tested as a control. A similar antidepressant effect was seen with physical cooling 6-7 weeks after stroke. Interestingly, we noticed that the antidepressant effect of physical cooling was weaker than that induced by HPI-363, implying the possibility that other effects of NTR1 activation, besides its hypothermic action, may also contribute to the anti-psychological benefits.

Impaired monoamine modulation is one of the major mechanisms underlying PSD. Previous studies showed that cerebral ischemia resulted in secondary degeneration of dopaminergic neurons in the midbrain, accompanied by reduced dopamine levels and DAT density [5]. In our study, reduced TH and DAT levels were found in the PFC at 8 weeks post stroke, indicating that focal ischemia might cause a chronic impairment on dopaminergic activity in the remote PFC. Serotonin is another key monoamine involved in depressive and anxious behaviors [11]. Although a significant change in the 5HT2AR expression in the PFC of stroke mice was not detected, more thorough examinations will be needed to determine whether the serotonin system was affected in non-ischemic regions after a focal ischemic stroke, particularly in brain areas associated with neuropsychological behaviors, such as the thalamus, hypothalamus and hippocampus.

Considering the increasing incidence of stroke with age, aging may raise more concerns in the treatment of post-stroke depression/anxiety. In addition, depressive elderly behaves and pharmacologically responses can be different from younger patients [66]. Therefore, further preclinic studies on pathogenesis or novel therapy of post-stroke depression/anxiety may pay more attention in aged animal models.

## Supplementary Materials

The Supplemenantry data can be found online at: www.aginganddisease.org/EN/10.14336/AD.2019.0507.
