# Supplementary material for: Neuropsychological Deficits Chronically Developed after Focal Ischemic Stroke and Beneficial Effects of Pharmacological Hypothermia in the Mouse
Source: Aging Dis. 2020 Feb 1;11(1):1–16. doi: 10.14336/AD.2019.0507 (PMC6961763; doi:10.14336/AD.2019.0507)
Supplement: Supplementary file 1 — The Supplemenantry data can be found online at: www.aginganddisease.org/EN/10.14336/AD.2019.0507. [file AD-11-1-1-s.pdf]

SUPPLEMENTARY DATA

Neuropsychological Deficits Chronically Developed after Focal Ischemic Stroke and Beneficial Effects of Pharmacological Hypothermia in the Mouse

Weiwei Zhong[1,2], Yan Yuan[1,3], Xiaohuan Gu[1,2], Samuel In-young Kim[1], Ryan Chin[1], Modupe Loye[2], Thomas A Dix[4], Ling Wei[1], Shan Ping Yu[1,2*]

[1]Department of Anesthesiology, Emory University School of Medicine, Atlanta, GA 30322, USA. [2]Center for Visual and Neurocognitive Rehabilitation, Atlanta Veterans Affairs Medical Center, Decatur, GA 30033, USA. [3]College of Veterinary Medicine, Yangzhou University, Yangzhou, 225009, China. [4]Department of Drug Discovery and Biomedical Sciences, Medical University of South Carolina, Charleston, SC 29401, USA.

© 2019. Zhong W et al. Published online at http://www.aginganddisease.org/EN/10.14336/AD.2019.0507

SUPPLEMENTARY DATA

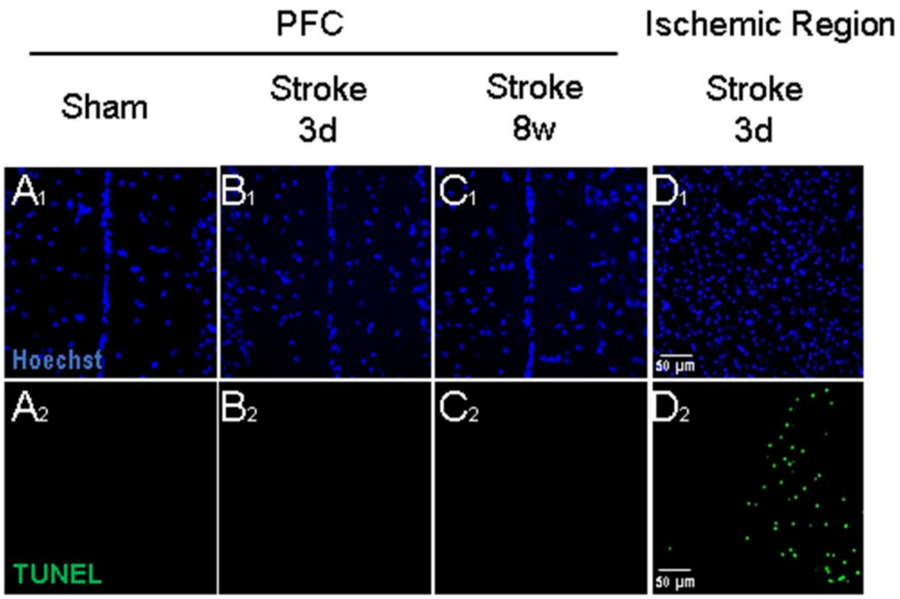

Supplementary Figure 1. TUNEL staining in the non-ischemic PFC after focal ischemia in the sensorimotor cortex. (A-C) No TUNEL-positive cells were detected in the PFC of sham and stroke mice. **(D)** TUNEL staining in the ischemic region was tested as positive control and TUNEL-positive cells were detected in the ischemic region at 3 days post stroke.

© 2019. Zhong W et al. Published online at http://www.aginganddisease.org/EN/10.14336/AD.2019.0507
